# Micro-mechanical investigation of railway ballast behavior under cyclic loading in a box test using DEM: effects of elastic layers and ballast types

**DOI:** 10.1007/s10035-019-0956-9

**Published:** 2019-10-24

**Authors:** Nishant Kumar, Bettina Suhr, Stefan Marschnig, Peter Dietmaier, Christof Marte, Klaus Six

**Affiliations:** 1 Virtual Vehicle Research Center, Inffeldgasse 21/A, 8010 Graz, Austria; 2 Institut für Eisenbahnwesen und Verkehrswirtschaft, Technische Universität Graz, Rechbauerstraße 12/II, 8010 Graz, Austria; 3 Institut für Baumechanik, Technische Universität Graz, Technikerstraße 4/II, 8010 Graz, Austria

**Keywords:** DEM, Railway ballast, Elastic layer, Under sleeper pad ($\text {USP}$), Under ballast mat ($\text {UBM}$), Conical damage model (CDM), Settlement, Edge breakage, Trackbed stiffness

## Abstract

**Abstract:**

Ballasted tracks are the commonly used railway track systems with constant demands for reducing maintenance cost and improved performance. Elastic layers are increasingly used for improving ballasted tracks. In order to better understand the effects of elastic layers, physical understanding at the ballast particle level is crucial. Here, discrete element method (DEM) is used to investigate the effects of elastic layers – under sleeper pad ($\text {USP}$) at the sleeper/ballast interface and under ballast mat ($\text {UBM}$) at the ballast/bottom interface – on micro-mechanical behavior of railway ballast. In the DEM model, the Conical Damage Model (CDM) is used for contact modelling. This model was calibrated in Suhr et al. (Granul Matter 20(4):70, 2018) for the simulation of two different types of ballast. The CDM model accounts for particle edge breakage, which is an important phenomenon especially at the early stage of a tamping cycle, and thus essential, when investigating the impact of elastic layers in the ballast bed. DEM results confirm that during cyclic loading, $\text {USP}$ reduces the edge breakage at the sleeper/ballast interface. On the other hand, $\text {UBM}$ shows higher particle movement throughout the ballast bed. Both the edge breakage and particle movement in the ballast bed are found to influence the sleeper settlement. Micro-mechanical investigations show that the force chain in deeper regions of the ballast bed is less affected by $\text {USP}$ for the two types of ballast. Conversely, dense lateral forces near to the box bottom were seen with $\text {UBM}$. The findings are in good (qualitative) agreement with the experimental observations. Thus, DEM simulations can aid to better understand the micro-macro phenomena for railway ballast. This can help to improve the track components and track design based on simulation models taking into account the physical behavior of ballast.

**Graphical Abstract:**

## Introduction

Classical railway tracks consist of a framework of rails and sleepers, which are supported on a compacted bed of ballast and sub-ballast that is laid on subgrade. Transmission of train loads from sleeper to the subgrade is considered to be one of the most important functions of railway ballast [2–5]. Ballast bed settles with regular loading from the moving train. Different settlement along the track results in track irregularities responsible for the dynamic responses of the vehicle, which must be kept within certain limits. Therefore, a better understanding of the ballast behavior and its interaction with track components is important, building the basis for significant improvements. Due to discrete nature of ballast stones, the discrete element method (DEM) is considered a suitable numerical tool to gain insight into the physical phenomena at the particle level and to simulate the bulk behavior of ballast. To correctly model the ballast behavior in DEM, it is important to determine the main physical phenomena occurring at the ballast scale. These phenomena should then be included in the contact law – the physical basis of DEM simulations – to capture as close as possible the short-term and long-term behavior of ballast across a range of experiments [6].

### Source for sleeper settlement

**Fig. 1:**
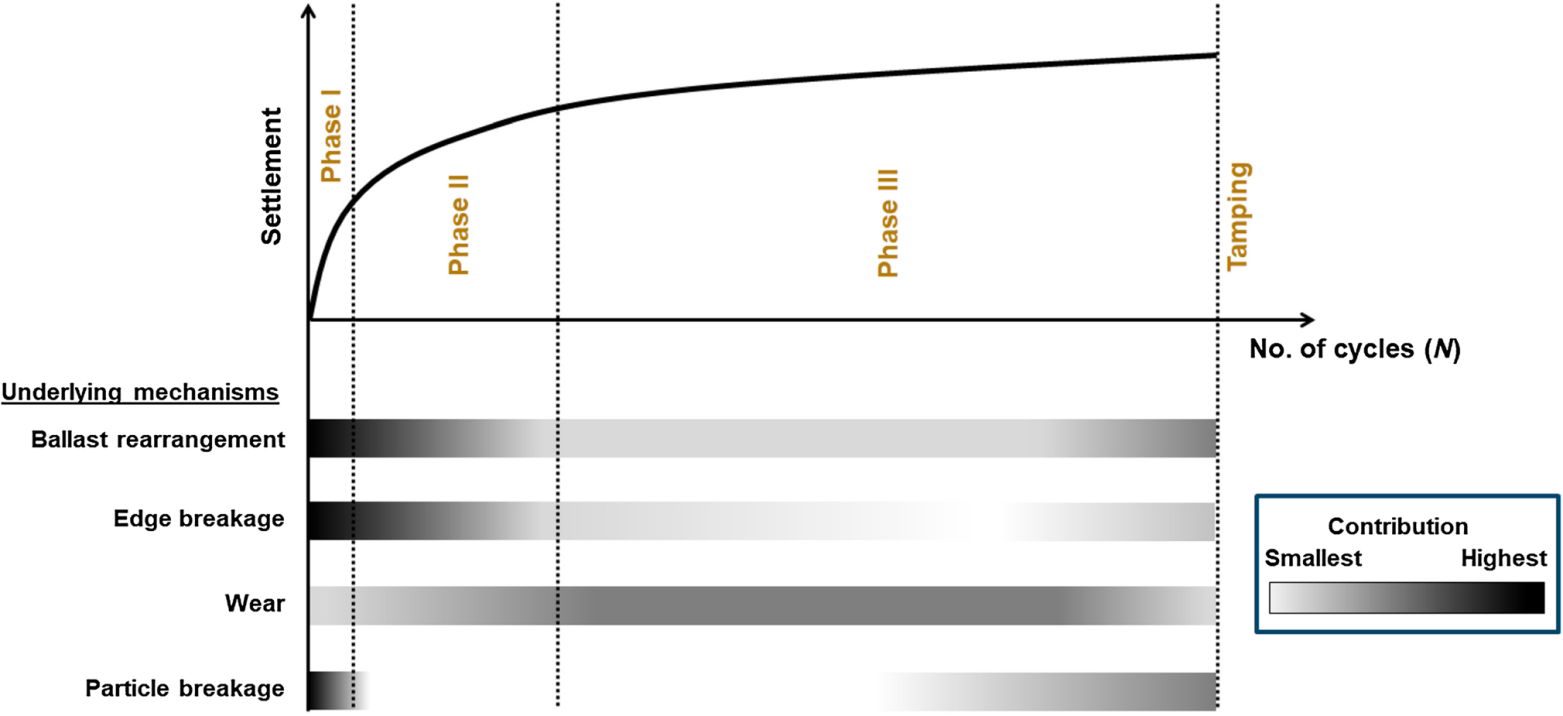
Sketch for sleeper settlement during a ballast tamping cycle. Ballast related phenomena contributing to sleeper settlement are ballast rearrangement, edge breakage, wear and particle breakage. The settlement cycle consists of three phases and these sources contribute differently in the three identified phases. Their contribution are highlighted in a gray scale: white and black means weak and strong contribution respectively

Based on literature survey, ballast rearrangement and ballast deterioration during the loading cycles are found to be one of the main sources responsible for ballast settlement, see [7–9] and reference therein. Ballast rearrangement occurs due to injection of energy by the moving vehicles, generally compacting the ballast bed. Few stones below the sleeper are responsible for the distribution of heavy loads to ballast bed that causes ballast deterioration, namely, edge breakage, wear and particle breakage [2, 5, 7, 10–13].

A settlement curve during a tamping cycle is sketched in Fig. 1 comprising of three different phases. Most importantly, it is anticipated that all the sources for ballast settlement contribute during the three phases, however, the contribution can be more dominant in one phase than in the other. In phase I, the settlement is relatively fast. Due to the fresh nature of the ballast bed, the settlement is dominated by ballast rearrangement (compaction as well as side-wise spreading) and breakage of the sharp edges of the stones [5, 7]. The largest voids that exist in the fresh ballast bed are reduced by several local particle rearrangements in this phase and settlement occurs at a fast rate [8]. Particle breakage may happen in this initial phase due to an awkward orientation of some of the ballast stones. In the “intermediate” phase II, ballast rearrangement is less due to already used up local voids in the previous phase. Thus, large-scale particle movements are necessary for small available void space, and settlement slows down [8]. By the end of phase II, edge breakage is considerably decreased, and the wear of rough ballast surface commences. In phase III, the settlement is slow and dominated by the wear of the ballast stones [9, 14]. Particle rearrangement and edge breakage rarely occur in phase III [7]. Near to the end of phase III, a state with ‘hanging sleeper’ may develop in the worst case. In this situation, the sleeper generates large impact forces, leading to significant ballast breakage and ballast rearrangement. Unavoidable differential settlement along the track at the end of phase III necessitates the next tamping cycle to restore the track geometry [15].

It is important to highlight that, not only settlement, but the track stiffness (mechanical strength of the bed) changes as well during a tamping cycle [16]. Both the settlement and track stiffness are important for characterizing a track; therefore, a good understanding of their evolution is crucial.

### Elastic layers in railway application

Elastic layers are being employed these days frequently in the railway tracks. Commonly used elastic layers include, under sleeper pads ($\text {USP}$) installed at the sleeper/ballast interface and under ballast mats ($\text {UBM}$) installed at the ballast/bottom interface. Elastic layers have shown to significantly reduce the ballast deterioration [10–13, 17, 18]. By installing a $\text {USP}$ under the sleeper, the track stiffness decreases. Thus, the rail tends to deflect more and the number of load bearing sleepers increase [16, 19]. This means the same load can be distributed to more sleepers and the peak load under a sleeper is reduced. The reduction in peak load reduces ballast deterioration, and, therefore results in better track performance [11].

### Literature overview

In the recent years, laboratory studies have been conducted to investigate the influence of elastic layers on the ballast behavior under cyclic loading. Berghold [10], Safari Baghsorkhi et al. [16], Abadi et al. [20] observed experimentally that under sleeper pads ($\text {USP}$) increase the contact area at the sleeper/ballast interface. Increase in contact area reduces the contact stress at this interface significantly [18, 20], which can be assumed to be the reason why $\text {USP}$ help to reduce ballast degradation, at least near the sleeper/ballast interface. This has been confirmed by the smaller amount of fines measured at the end of test with $\text {USP}$ [12, 13, 18]. Not only the contact area, but the number of contacts at the sleeper/ballast interface also increase with $\text {USP}$ [20]. A common behavior observed by most researchers in experimental tests is the reduced settlement with $\text {USP}$ [10, 12, 16, 18, 21], however a clear understanding at the microscopic ballast level scale is experimentally challenging. In track systems, due to higher bending of the rail with $\text {USP}$, the axle load is distributed to more sleepers. Therefore, it is expected that the reduction in settlement is even more pronounced with $\text {USP}$ due to reduction in peak loads. Safari Baghsorkhi et al. [16] showed that the stiffness of the single sleeper-ballast assembly is smaller with $\text {USP}$. In terms of the stress transfer to the bottom, Berghold [10] and Abadi et al.[20] observed no significant influence of $\text {USP}$ on the pressure measured at the bottom. In fact, Abadi et al. [20] found very little variation in the bottom pressure for different ballast gradation and variety of sleepers used. Regarding the effects of the elastic layer at the bottom $\text {UBM}$, Raymond and Bathurst [22] performed cyclic loading tests for three different bottom stiffnesses and showed that the ballast settlement increases with decreasing bottom stiffness. Higher ballast degradation was observed for the soft bottom, possibility due to increase in aggregate movements with decreasing support stiffness. Concerning different ballast types, Berghold [10] observed that the ballast susceptible to higher degradation show higher settlement. Although several effects of elastic layers have been observed in laboratory tests, a good understanding at the microscopic ballast scale still remains unclear.

The discrete element method (DEM) based modelling of railway ballast has achieved significant progress in the last decades. A reason is the method’s ability to provide insight into different phenomena occurring at the particle scale. Accurately modelling the irregular ballast shape using clumps of discs/spheres in DEM has been investigated in [2–4, 23–28]. Compared to single sphere, irregular particle shape provides more particle interlocking, with reduced rotations, mimicking realistic load-deformation response [23]. Ballast degradation mechanisms like edge breakage and particle breakage has been modeled as well using bonded particles [2, 4, 23, 27, 29]. A major problem with modelling ballast degradation with DEM is dealing with large number of small sized fines, which increases the computational time. This limits the validation of DEM results against experiments. Modelling the influence of elastic layers on ballast behavior using DEM was studied recently [4]. Li and McDowell [4] modeled the $\text {USP}$ in detail with bonded sphere, and showed that $\text {USP}$ increases the number of contacts points at the sleeper/ballast interface and decreases the sleeper settlement. It was found that loads are distributed laterally with $\text {USP}$ which reduces the settlement.

Major focus has been put to correctly model the ballast shape in the DEM model, and only recently, it came to light that the choice of contact model for the railway ballast plays an important role for capturing correctly the experimental observations by consideration of physical phenomena [1, 30, 31]. The authors showed that Hertz–Mindlin contact law with a single set of parameters, at least for a simplified ballast DEM geometry, cannot capture the bulk behavior of ballast for e.g.,  monotonic and triaxial tests [30], and, compression and direct shear tests [1, 31]. This led to the development of the Conical Damage Model (CDM) accounting for edge breakage of ballast. Suhr et al. [1] calibrated the CDM model for two types of ballast, Kieselkalk and Calcite. Their model and the calibrated material parameters (with enlarged particles) will be used in this study.

Effects of elastic layers on mirco–marco behavior of two types of ballast, Kieselkalk and Calcite, will be investigated in this study. The strength of the CDM model will be evaluated in this work by (qualitatively) comparing the results for cyclic loading for 200 cycles, with observations from literature on railway ballast. Referring to phases of ballast settlement presented in Fig. 1, the present study is considered to be in the phase I and early phase II, where ballast rearrangement and edge breakage are the dominating sources for sleeper settlement. Both these physical mechanisms are included in the DEM model in this study, which was not possible with the simple Hertz–Mindlin contact law that can account for ballast rearrangement only. The paper is organized as follows. Section 2 discusses the CDM model for railway ballast and the material parameters for different components. Parameters for the box, sleeper and elastic layers ($\text {USP}$ and $\text {UBM}$) are also discussed there. The various stages in the preparation of the ballast bed and the cyclic loading experiments are presented in Sect. 3. Effects of elastic layers on the contact area at the sleeper/ballast interface and evolution in contact number with loading cycles is presented in Sect. 4. The sleeper settlement and the micro-mechanical mechanisms responsible for sleeper settlement as well as effects of elastic layers and types of ballast on the system stiffness and bottom pressure, are also presented in this section. Finally, conclusions and outlook are drawn in Sect. 5.

## DEM model

In this work, a recently developed conical damage model (CDM) for particle–particle contact of railway ballast will be used in DEM simulations, that accounts for the edge breakage of the ballast under high stresses [1, 30, 31]. The model is described as follows: in the normal contact direction, an elastic regime exists, which is modeled with Hertzian law. When a certain stress is exceeded, a kind of ideal plasticity is introduced to model damage at the contact. In this phase, the surface of the DEM particle is imagined to flatten locally due to damage (particle geometry in DEM remains unchanged). The flattening corresponds to a larger surface area and thus to a lower stress. In tangential direction, the classical Mindlin law together with a constant coefficient of friction is used. The reader is referred to the original work for detailed description of the CDM model equations and the used algorithms in [1, 31]. The CDM model has five parameters. The Young’s modulus *E*, the Poisson ratio $\nu$, the interparticle friction coefficient $\mu$, are the three parameters same as in the classical Hertz–Mindlin model. Two additional parameters are pseudo maximal compressive strength $\sigma _{\max }$, and $\beta$ which relates to the part of contact overlap associated with plastic yielding. It is important to mention that in the present state of the DEM modelling, contact yielding (edge breakage) history is saved only as long as a certain contact is active. For the investigated cases, only few contacts get lost and become active again during the load cycles.

**Fig. 2:**
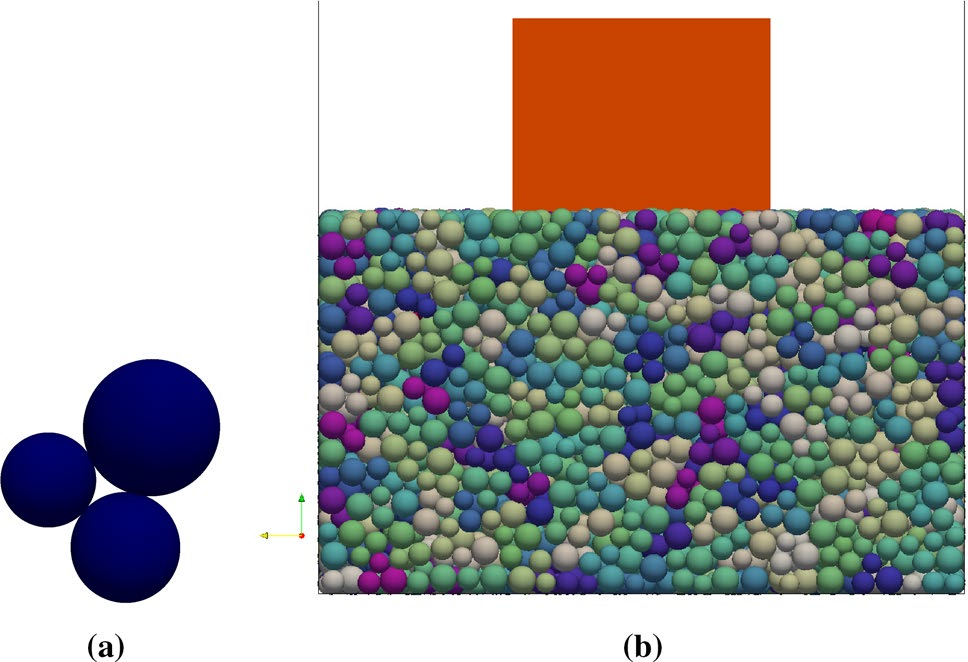
**a** Clump formed by three non-overlapping, unequal sized spheres used as a ballast particle in the DEM simulations. **b** Snapshot of the system including the sleeper after the two preparation stages. Spheres of same clump are shown with one color, while colors of the clump has no meaning (color figure online)

Two different types of ballast Kieselkalk and Calcite are considered in this study. The corresponding CDM model parameters with simple clump shapes for the two types of ballast were calibrated by Suhr et al. [1]. A short summary of the experiments and the calibration process is given here, and the reader is referred to the original work for details. Suhr conducted compression and direct shear box experiments on the two types of ballast. The shear box itself had the size 300 mm $\times$ 300 mm $\times$ 200 mm and was divided horizontally at medium height. The initial porosity was around 0.45 for the two samples. In the compression test, the loads were applied from 10 to 30 kN (corresponding to stress from 111 to 333 kPa) for four cycles using a steel plate covering the top of ballast. It was observed that the stiffness (slope of force–displacement curve) of Kieselkalk is lower than Calcite during the compression test. For the direct shear test, three different values of normal load were chosen 10 kN, 20 kN and 30 kN (corresponding stress values 111 kPa, 222 kPa and 333 kPa) and direct shear tests were conducted for each level of the applied normal loads. The results of direct shear test showed surprisingly similar results for both types of ballast. Compared to Kieselkalk, it was observed that Calcite generated a slightly higher amount of fines. A DEM model using simple clump shapes using the CDM contact law was built. Same experiments were performed in the DEM simulations and *one set* of parameters was established for each type of ballast. The obtained DEM simulation results were in good accordance with the experiments which was not possible with simple Hertz–Mindlin contact law. The parameters for both types of ballast are specified in Table 1. Suhr et al. [1] used a ballast shape formed by a clump of three non-overlapping spheres with different radii, 7 mm, 5.8 mm and 5 mm, placed in a triangle. Here, the spheres radii are increased by 1.5625 to achieve a clump major axis of 40 mm. This allowed for less particles used in a simulation and this is the recommended $d_{50}$ ballast particle size in the railway operation [32]. All clumps have the same size; thus no gradation/size distribution of the ballast is considered. A DEM particle representation of ballast is shown in Fig. 2a.

The cyclic loading simulations are conducted in a steel box. Detailed modelling of the elastic layers in DEM is beyond the scope of this study, and they are simply modeled with a reduced Young’s modulus *E* of their stiff counterparts (concrete sleeper and stiff bottom), as seen in Table 1. Both the box and the sleeper are elastic in nature, and the plastic deformation of these components is not considered in the DEM modelling. To avoid any confusion with a meticulous modelling of elastic layers, under sleeper pad ($\text {USP}$) at the sleeper/ballast interface will be denoted as $\text {USP}_{\text {EL}}$ and under ballast mat ($\text {UBM}$) at the ballast/bottom interface will be denoted as $\text {UBM}_{\text {EL}}$ throughout this article. The DEM properties of the steel box, concrete sleeper and elastic layers are specified in Table 1. The idea is to present, even with a simplistic modelling of elastic layers, the strength of a careful modelling of the physical phenomena at the particle level scale. It can explain several experimental observations, thus, building the basis for significant improvements of the track system.

**Table 1:** Material parameters used in the simulation

	*E* (GPa)	$\nu$ (–)	$\mu$ (–)	$\sigma _{\max }$ (MPa)	$\beta$ (–)	$\rho$ (kg/m$^3$)
*Ballast properties*						
Demo ballast	3	0.2	0.0	200	0.0154	2600.0
Kieselkalk	30	0.2	0.45	280	0.0098	2660.0
Calcite	60	0.2	0.45	600	0.0154	2822.2
*Wall properties*						
Steel box	200	0.28	0.2	–	–	–
$\text {UBM}_{\text {EL}}$	0.02	0.28	0.2	–	–	–
*Sleeper properties*						
Concrete sleeper	52.409	0.167	0.7	–	–	2749.8
Concrete sleeper with $\text {USP}_{\text {EL}}$	0.052409	0.167	0.7	–	–	2749.8

**Table 2:** Six different test cases considered in this study. The material properties of components are given in Table 1

Case	Ballast	Wall	Sleeper
I	Kieselkalk	Steel box	Concrete Sleeper
II	Kieselkalk	Steel box	Concrete sleeper with $\text {USP}_{\text {EL}}$
III	Kieselkalk	Steel box with $\text {UBM}_{\text {EL}}$	Concrete sleeper
IV	Kieselkalk	Steel box with $\text {UBM}_{\text {EL}}$	Concrete sleeper with $\text {USP}_{\text {EL}}$
V	Calcite	Steel box	Concrete Sleeper
VI	Calcite	Steel box	Concrete sleeper with $\text {USP}_{\text {EL}}$

## Simulation setup

The DEM simulations are done using software YADE [33] which is Open-Source and utilizes the soft contact approach together with explicit integration in time. Due to its openness, the implementation of new or adapted contact laws is possible, whenever needed. Since careful, well-defined sample preparation is essential in any physical experiment to obtain reproducible results [34], the preparation procedure is discussed in detail next. The preparation of the ballast bed consists of two stages. In the stage (1), a relaxed, dense, and evenly surfaced ballast bed is generated, and this is done only once. To reduce the variations due to preparation step, the generated sample from stage (1) is used to prepare different test cases containing sleeper in stage (2) (described in Table 2).

### Preparation stage (1)

The preparation stage (1) consists of four parts: (i) gravity settling, (ii) vibration, (iii) smoothing of the top surface, and, (iv) relaxation. ‘Demo’ ballast particles were used in this stage, which are soft in nature and have no friction for generating dense samples. The properties of the demo ballast are changed later to the corresponding ballast after the preparation stage (1). This approach has two benefits: firstly, it permits to choose a larger time step in DEM simulations and saves considerable computation time, and, secondly, it helps in reducing the statistical variations in distribution of particle arrangement due to preparation procedure, allowing for an acceptable comparison across different cases. A ballast particle is shown in Fig. 2a, and the material parameters used in the simulations are specified in Table 1. A loose cloud of 4300 monodisperse demo ballast (roughly 13,000 spheres) was created randomly in a 0.5 m $\times$ 0.5 m $\times$ 2.0 m steel box that settled under gravity. When the kinetic energy per particle reached below $10^{-5}$ J, a vibration was applied from the bottom. This was done to inject energy into the system, allowing the particles to find a better position in the ballast bed, inducing further compaction of the generated bed. The vibration was applied for three cycles with an amplitude of 2 mm and frequency 40 Hz, with intermediate relaxation for 0.25 s. Afterwards, a plate was inserted above the topmost ballast stone to smooth the top surface. The plate moved downwards with a constant velocity $v_{\text {plate}}=0.01$ m/s. When the plate had moved downwards by 20% of the initial bed height, the velocity of the plate was reversed. The plate was deleted from the scene when it had no more contact with the ballast bed. Followed by a relaxation for 1.0 s, a relaxed, dense, and even surfaced ballast bed was achieved.

### Preparation stage (2)

In this stage, the sample generated in the previous stage is used. Initially, the material properties of the ballast and wall were changed to corresponding cases, namely: Kieselkalk in steel box, Kieselkalk in steel box with $\text {UBM}_{\text {EL}}$, and Calcite in steel box, and the new set of samples were relaxed for 0.5 s. Then, a plate was inserted above the highest particle in the bed which moved downward with a constant velocity $v_{\text {plate}}=0.01$ m/s until the maximum force on the plate was $F_{\text {plate}}=83.25$ kN (corresponding to a pressure of $P_{\max }=333$ kPa). The velocity of the plate was then reversed, and it moved upward till the force on the plate is zero. This process was repeated for three cycles and the plate is removed from the scene afterwards. All the ballast particles above the height $h_{\text {bed}}=0.30$ m were deleted and the samples were relaxed further for 0.5 s. Thus, the ballast bed for the three test cases are realized, and each have a porosity $\phi =0.40$, containing $\approx 4200$ clumps.

In the final phase of the preparation step, a sleeper was added above the ballast bed for performing the cyclic loading experiments. Two different types of sleeper are used – concrete sleeper and sleeper with $\text {USP}_{\text {EL}}$ – with properties specified in Table 1. A cuboid sleeper with dimensions 0.2 m $\times$ 0.2 m $\times$ 0.15 m was generated in the center, just above the ballast bed, that falls under gravity. When the kinetic energy of the sleeper reached below $10^{-7}$ J, an external consolidation pressure is applied on the ballast bed through the sleeper. The consolidation due to the weight of the sleeper and external pressure is $P_{\min }=111$ kPa, and the particles on the free surface have no pressure. A final relaxation for 0.5 s was performed in the end. At this point, the time is reset to zero and the displacements of different components in the later section will be defined relative to their position after this final preparation stage. Thus, after the two parts of the preparation stage, six different cases reported in Table 2 are created which undergo cyclic loading experiments. The final ballast bed including the sleeper is shown in Fig. 2b.

### Main experiment:

For the main experiments, on the top of constant consolidation pressure $P_{\min }$, external (force) pressure is applied on the sleeper that transfers this load onto the ballast bed. The applied external pressure over time *t* has the form of a smooth cosine function:

1 $$\begin{aligned} P_{\text {ext}}=0.5 \left( P_{\max }- P_{\min }\right) \left( 1 - \cos \left( 2\pi f t \right) \right) , \end{aligned}$$

where, $P_{\min }=111$ kPa is the constant consolidation pressure on the ballast, $P_{\max }=333$ kPa is the maximum pressure on the ballast, and $f=3$ Hz is the frequency of the applied cyclic load. This is the pressure range applied in the experimental and numerical works in Refs. [1, 4, 10, 16, 18, 20, 22], allowing for comparison at different stages of this study. The chosen cosine functional form of the external load enables a smooth cyclic loading. Interestingly, it is quite close to the applied load due to a moving vehicle in real track conditions and will be discussed in another paper. The low loading frequency $f=3$ Hz is chosen to keep the dynamic effects low in the system, and the same external loading path given by Eq. 1 is applied to all the six cases discussed in Table 2. It is important to mention that the sleeper has only one vertical degree of freedom in the *z*-direction, and its motion in *x*, *y*-direction is restricted, as well as the rotations.

## Results

In this section, the micro–macro behavior of ballast bed is investigated to study the effects of (i) elastic layers at the sleeper/ballast interface $\text {USP}_{\text {EL}}$, (ii) elastic layers at the ballast/bottom interface $\text {UBM}_{\text {EL}}$, and (iii) ballast types (Kieselkalk and Calcite) during 200 loading cycles.

### Sleeper ballast contact area

**Fig. 3:**
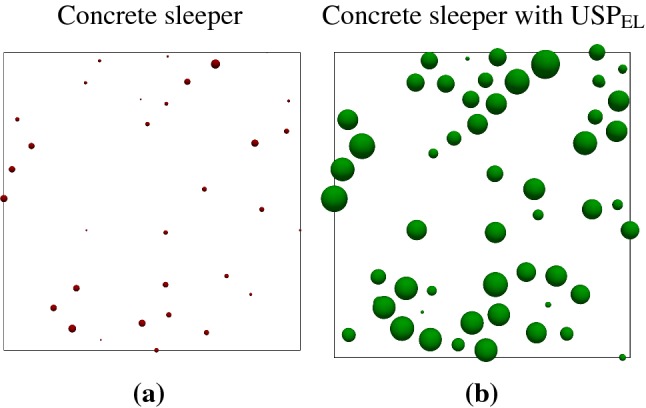
Contact area at the sleeper/ballast interface at the maximum load at the $200{\text {th}}$ cycle for Kieselkalk in steel box for **a** concrete sleeper and **b** concrete sleeper with $\text {USP}_{\text {EL}}$

Figure 3 shows the contact area at the sleeper/ballast interface for Kieselkalk ballast in a steel box, with and without elastic layer $\text {USP}_{\text {EL}}$ at the sleeper/ballast interface (cases I and II in Table 2). The contact area is presented at the maximum load at the $200{\text {th}}$ cycle. Compared to concrete sleeper, $\text {USP}_{\text {EL}}$ shows higher sleeper/ballast contact area. In this study, soft nature of $\text {USP}_{\text {EL}}$ is modeled with a reduced Young’s modulus *E* by four orders than the concrete sleeper, leading to a much smaller effective Young’s modulus $E_{\text {eff}}$ of the contacts at the sleeper/ballast interface. Thus, a sleeper with $\text {USP}_{\text {EL}}$ tend to penetrate more (higher overlap) into the ballast particle, and is the reason for higher contact area with $\text {USP}_{\text {EL}}$. This observation is in agreement with the experimental work of Refs. [18, 20], where higher sleeper/ballast contact area is observed with $\text {USP}_{\text {EL}}$ (cumulative contact area over many cycles). When an elastic layer $\text {UBM}_{\text {EL}}$ is used at the bottom, higher contact area with $\text {USP}_{\text {EL}}$ was observed as well. Moreover, for the Calcite ballast, higher contact area with $\text {USP}_{\text {EL}}$ was seen as well, and therefore not shown.

### Number of contacts

**Fig. 4:**
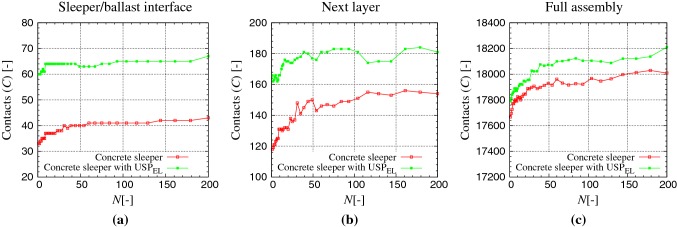
Number of contacts versus number of load cycles: ballast contacts with **a** sleeper, **b** next layer (defined as ballast–ballast contacts, where one contact partner is also in contact with the sleeper) and **c** in the full assembly, for Kieselkalk in steel box. Red and green data sets represent results for the concrete sleeper and concrete sleeper with $\text {USP}_{\text {EL}}$ respectively (color figure online)

**Table 3:** Table summarizing the average force in different sections of the ballast bed and net stiffness $k_{{}}$ for the six different test cases considered in this study. The different cases are presented in Table 2. The average force is measured at the peak load during the $200{\text {th}}$ cycle

Case	Average force (N)			
	Sleeper/ballast interface	Ballast/bottom interface	Total assembly	Stiffness $k_{{}}$ (MPa/m)
I	320	38	25	2580
II	200	38	25	330
III	270	21	25	1640
IV	190	21	25	320
V	360	38	25	3240
VI	235	38	25	305

In this section, the ballast bed is examined microscopically to investigate the effects of $\text {USP}_{\text {EL}}$. Particle (sphere) contacts are analyzed in three different sections of bed: sleeper/ballast interface, next layer contacts (defined as ballast–ballast contacts, where one contact partner is also in contact with the sleeper), and all the contacts in the system. Fig. 4 shows the evolution of contacts with cycles *N*, at the maximum load, for Kieselkalk in steel box, with and without $\text {USP}_{\text {EL}}$ (cases I and II from Table 2), in the different sections of the bed. In all the three analyzed sections of the assembly, higher number of contacts can be seen with $\text {USP}_{\text {EL}}$. After approximately 50 cycles, a saturation in the number of contacts in the three sections can be seen for both types of sleeper. At the sleeper/ballast interface shown in Fig. 4a, the number of contacts with $\text {USP}_{\text {EL}}$ is higher than without by 1.6 times (or 25 more contacts). Soft nature of $\text {USP}_{\text {EL}}$ allows the sleeper to go deeper in the ballast bed, thus it has the possibility to have higher number of ballast contacts. At this interface, higher number of contacts lead to smaller average force per contact with $\text {USP}_{\text {EL}}$. At the maximum load, the average normal force per force bearing contact at the sleeper/ballast interface is 200 and 320 N, with and without $\text {USP}_{\text {EL}}$, respectively.

The next ballast layer also has more contacts with $\text {USP}_{\text {EL}}$ (30 more contacts), as shown in Fig. 4b. This is again due to the deeper (compression) penetration of the sleeper in the ballast bed with $\text {USP}_{\text {EL}}$. When looking at the contacts in the full assembly in Fig. 4c, system with $\text {USP}_{\text {EL}}$ shows higher (around 100 more) number of contacts. This is mainly due to difference in number of contacts near the sleeper. Thus, regarding the ballast contacts, mostly the region near the sleeper is affected by using $\text {USP}_{\text {EL}}$, while the rest of the ballast bed is barely affected. At the maximum load, the average normal force per force bearing contact in the total assembly is 25 N for both systems with and without $\text {USP}_{\text {EL}}$. DEM simulations showed similar observations for the ballast contacts in the three different sections of the bed, when the elastic layer $\text {UBM}_{\text {EL}}$ is used at the bottom and for the Calcite ballast, thus not presented. Table 3 summarizes the average force in different sections of the ballast bed for different cases.

Comparing the DEM observation with literature, Abadi et al. [20] measured the ratio of contacts between the concrete sleeper and softest $\text {USP}$ at the sleeper/ballast interface to be 3, while the ratio was 1.6 in this work. This difference can be because of (i) simple ballast shape used for the DEM model in this work, and/or (ii) cumulative contacts were counted after the finish of millions of loading cycles in [20], while here they are counted at the maximum load at the $200{\text {th}}$ cycle. The agreement, though, is that the $\text {USP}$ increases the number of sleeper/ballast contacts.

### Sleeper settlement under cyclic loading

**Fig. 5:**
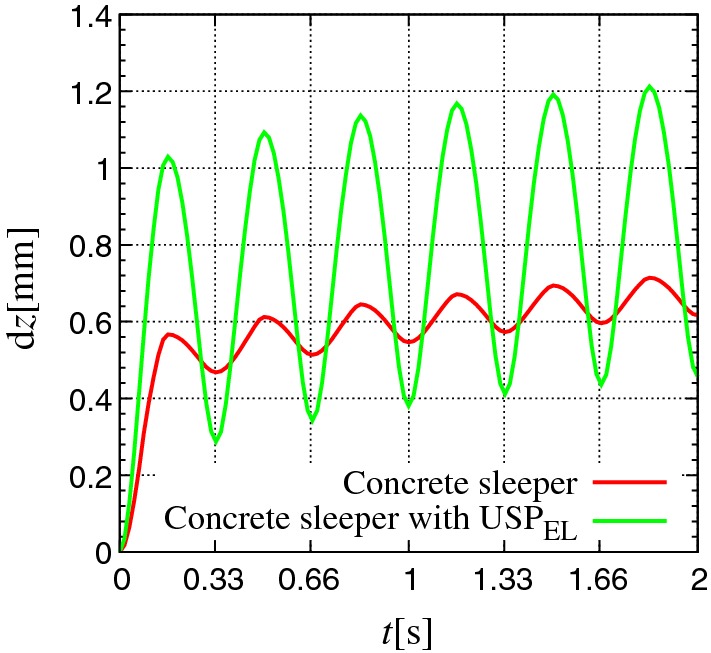
Position of sleeper with time over 6 cycles for Kieselkalk in steel box (cases I and II in Table 2). Red and green data sets represent results for the concrete sleeper and concrete sleeper with $\text {USP}_{\text {EL}}$ respectively (color figure online)

**Fig. 6:**
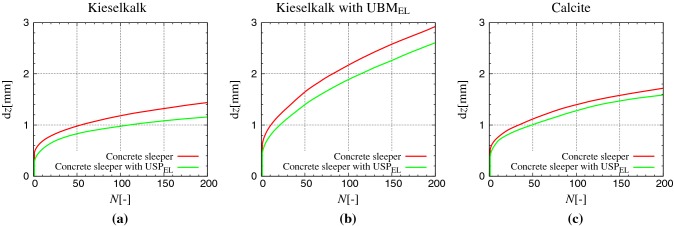
Settlement of sleeper with cycles *N* for the three samples **a** Kieselkalk in steel box, **b** Kieselkalk in steel box with $\text {UBM}_{\text {EL}}$, and **c** Calcite in steel box. Red and green data sets represent results for the concrete sleeper and concrete sleeper with $\text {USP}_{\text {EL}}$ respectively. For comparison purposes, same axes are used for all cases (color figure online)

Figure 5 shows the sleeper displacement (with respect to initial position $\mathrm {d}z$) with time *t*, over the first six loading cycles for Kieselkalk in steel box, with and without $\text {USP}_{\text {EL}}$. When the load $P_{\text {ext}}$ increases, the sleeper goes inside the ballast bed and does not return to its original position after unloading, when the external load $P_{\text {ext}}$ is zero. This process continues with further loading cycles and a “plastic” displacement (irrecoverable sleeper displacement) of the sleeper can be seen with each load cycle. After the first cycle, the sleeper max–min displacement during unloading with and without $\text {USP}_{\text {EL}}$ is $\approx$ 0.78 mm and $\approx$ 0.10 mm respectively. Higher sleeper displacement with $\text {USP}_{\text {EL}}$ can be explained by the smaller effective Young’s modulus $E_{\text {eff}}$ of contacts at the sleeper/ballast interface with $\text {USP}_{\text {EL}}$. Therefore, for the same applied external load, the displacement of the sleeper into the ballast bed is higher with $\text {USP}_{\text {EL}}$. Higher sleeper displacement with $\text {USP}_{\text {EL}}$ during loading, and irrecoverable sleeper displacement with every cycle with and without $\text {USP}_{\text {EL}}$ was also observed when an additional elastic layer at the bottom $\text {UBM}_{\text {EL}}$ was introduced. DEM simulations showed similar observations for the Calcite ballast with and without $\text {USP}_{\text {EL}}$, and therefore not shown.

Data points for sleeper displacement after the end of each cycle in Fig. 5 when connected, gives the sleeper settlement $\mathrm {d}z$ with cycles *N*. $\mathrm {d}z$ increases monotonically (accumulated irrecoverable displacement) with loading cycles *N*. Fig. 6 shows the settlement of sleeper $\mathrm {d}z$ with cycles *N*, for all cases presented in Table 2 that have the same initial particle configuration. The following observations can be made for:

(i) elastic layers at sleeper/ballast interface: sleeper settlement with $\text {USP}_{\text {EL}}$ is smaller for all the three systems: Kieselkalk in steel box, Kieselkalk in steel box with $\text {UBM}_{\text {EL}}$, and Calcite in steel box, shown in Fig. 6;
(ii) elastic layers at ballast/bottom interface: Kieselkalk in steel box with $\text {UBM}_{\text {EL}}$ shows higher sleeper settlement compared to Kieselkalk in steel box, shown in Fig. 6a, b;
(iii) different ballast types: Calcite shows slightly higher sleeper settlement compared to Kieselkalk in steel box, shown in Fig. 6a, c.

In the next section, further microscopic investigations are performed to identify the dominant source(s) for the different sleeper settlement observed for different cases.

#### Microscopic quantities

In the presented DEM modelling of railway ballast, particle edge breakage using CDM model, and particle movement due to cyclic loading, are the two possible mechanisms responsible of sleeper settlement^1^. In order to identify the contribution of these two mechanisms at the microscopic ballast scale, towards the different sleeper settlement observed for six cases in Fig. 6, microscopic quantities are defined in this section.

To quantify the degree of edge breakage, fraction of yielding contacts $Y_{{}}$ is defined as:

2 $$\begin{aligned} Y_{{}}=\frac{M^{\text {yl}}_{{}}}{M_{{}}}, \end{aligned}$$

where $M^{\text {yl}}_{{}}$ and $M_{{}}$ represent the instantaneous number of yielding contacts and total contacts respectively, in an observation volume. A yielding contact (contact with edge breakage) has normal force $F_N>0$ and normal stress $\sigma = \sigma _{\max }$. $Y_{{}}$ can take values between 0 and 1; 0 for no yielding contacts, and 1 when all the contacts are yielding in a representative volume. Note that in the present state of the CDM model, contact yielding (edge breakage) history is saved as long as the a contact remains active. $Y_{{}}$ values calculated from contacts at the sleeper/ballast interface and in the full assembly will be used in this work.

Both the compaction of the bed under the sleeper and side-wise spreading of the ballast contributes to sleeper settlement [8]. The two effects can be merged into a single quantity $\chi$, quantifying the average particle movement in the ballast bed, defined as:

3 $$\begin{aligned} \chi =\left\langle {\mathbf {r_p}(t) - \mathbf {r_p}(t=0) } \right\rangle , \end{aligned}$$

where $\mathbf {r_p}(t)$ is the vector position of (sphere) particle *p* in an observation volume at time *t*. $\mathbf {r_p}(t=0)$ is the vector position of the corresponding particle at the beginning of cyclic loading at $t=0$. $\chi$ includes the displacement of each sphere, thus, the rotation of ballast (clump of three spheres shown in Fig. 2a) is also included in $\chi$. By definition $\chi$ is a positive number and higher $\chi$ means higher particle movement. $\chi$ values calculated near the sleeper (all spheres within a *z*-distance 4 times maximum particle diameter from the sleeper) and in the full assembly will be used in this work.

#### Microscopic investigation

**Fig. 7:**
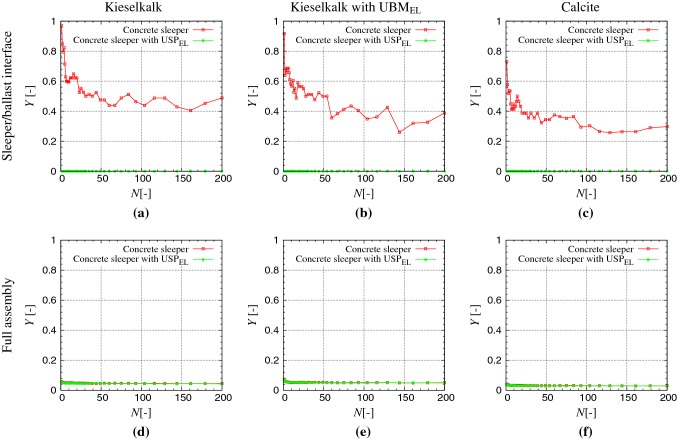
Fraction of yielding contacts $Y_{{}}$ plotted against loading cycles *N*, at the peak load at the sleeper/ballast interface (top row) and in the full assembly (bottom row), for the three samples: Kieselkalk in steel box (first column), Kieselkalk in steel box with $\text {UBM}_{\text {EL}}$ (second column), and Calcite in steel box (third column). Red and green data sets represent results for the concrete sleeper and concrete sleeper with $\text {USP}_{\text {EL}}$ respectively. For comparison purposes, same axes are used for all cases (color figure online)

Figure 7 (left column) show the fraction of yielding contacts $Y_{{}}$ with cycles *N*, at the maximum load, for two observation sections of ballast bed: at the sleeper/ballast interface, and in the full assembly, for Kieselkalk in steel box, with and without $\text {USP}_{\text {EL}}$ (cases I and II in Table. 2). After 100 cycles, a saturation in the $Y_{{}}$ value can be seen. With concrete sleeper, almost 40% the contacts at the sleeper/ballast interface are yielding; i.e. $\sigma = \sigma _{\max }$ with $Y_{{}}\approx 0.40$, as shown in Fig. 7a. On the other hand, with $\text {USP}_{\text {EL}}$, no edge breakage (no yielding) at the sleeper/ballast interface is observed, i.e., $Y_{{}}=0$. $Y_{{}}=0$ is possible when the normal contact force $F_N$ is small, and/or the contact area is large, such that the contact stress $\sigma < \sigma _{\max }$. With $\text {USP}_{\text {EL}}$, higher number of contacts at the sleeper/ballast interface was seen in Fig. 4a. This means that the average force per contact at this interface is small with $\text {USP}_{\text {EL}}$, since the same loading path is applied to all the different cases. Furthermore, contacts at this interface show higher contact area with $\text {USP}_{\text {EL}}$ seen in Fig. 3. Both the two conditions, smaller contact force and higher contact area with $\text {USP}_{\text {EL}}$, lead to less contact stress $\sigma < \sigma _{\max }$, and therefore less edge breakage at the sleeper/ballast interface observed with $\text {USP}_{\text {EL}}$ in Fig. 7a. Contrary to the difference in $Y_{{}}$ at the sleeper/ballast interface, in the full assembly, $Y_{{}}$ is found to be approximately the same for both cases, with and without $\text {USP}_{\text {EL}}$, as shown in Fig. 7d. It is important to mention again, that in the present study, the two systems with and without $\text {USP}_{\text {EL}}$, undergo the same loading paths. In the real track system, peak loads are reduced with $\text {USP}_{\text {EL}}$, therefore ballast degradation is expected to be less in the full assembly as well with $\text {USP}_{\text {EL}}$.

Regarding the effect of the elastic layer at the bottom, $\text {UBM}_{\text {EL}}$ has no significant influence on $Y_{{}}$ at the sleeper/ballast interface, as shown in Figs. 7a, b. Due to the soft nature of $\text {UBM}_{\text {EL}}$, no edge breakage at the box bottom is observed, i.e., $Y_{{}}=0$ compared to $0.6\%$ edge breakage observed for steel bottom (data not shown). In the full assembly with $\text {UBM}_{\text {EL}}$, $Y_{{}}$ is higher by $0.5\%$. This behavior of higher edge breakage with $\text {UBM}_{\text {EL}}$ is in agreement with the experimental work by Raymond and Bathurst [22], where higher fines were recovered with soft bottom after the cyclic loading test. The explanation given in [22] for the higher ballast degradation with $\text {UBM}_{\text {EL}}$, is the (expected) higher ballast movements with soft bottom. For the two types of ballast, $Y_{{}}$ with cycles *N* for Kieselkalk and Calcite are plotted in Fig. 7 in the first and third columns, respectively. At the sleeper/ballast interface, high edge breakage is observed without $\text {USP}_{\text {EL}}$, while no edge breakage at this interface was observed with $\text {USP}_{\text {EL}}$ for both types of ballast. Edge breakage in the full assembly is approximately the same for the two types of ballast.

**Fig. 8:**
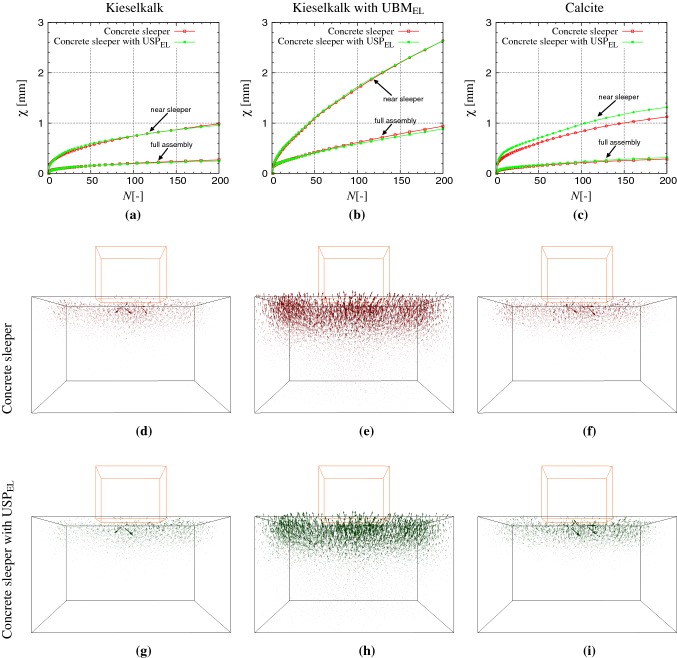
(Top row) Ballast movement $\chi$ plotted against loading cycles *N* at the peak load in the full assembly for the three samples: Kieselkalk in steel box (first column), Kieselkalk in steel box with $\text {UBM}_{\text {EL}}$ (second column), and Calcite in steel box (third column). Red and green data sets represent results for the concrete sleeper and concrete sleeper with $\text {USP}_{\text {EL}}$ respectively. $\chi$ near the sleeper (calculated using all spheres within a *z*-distance 4 times maximum particle diameter from sleeper) and in the full assembly is shown by circles and squares respectively. For comparison purposes, same axes are used for all cases. Screenshots of particles (sphere) displacement at the peak load at the $200{\text {th}}$ cycle for systems with concrete sleeper (middle row) and concrete sleeper with $\text {USP}_{\text {EL}}$ (bottom row) for the three samples are shown in **d**–**i**. Arrow length is proportional to the magnitude of the displacement

In Fig. 8 (top row), particle movement $\chi$, as defined in Eq. 3, is plotted with cycle *N* at the peak load for all the six cases. Particle movement $\chi$ increases with the loading cycle *N* for all cases. This is because of the expected irreversible plastic displacement of (sphere) particles with every large-strain loading cycle, i.e., particles move during loading and do not come back to their original position after unloading [35]. Movement of particles close to the sleeper contributes most to $\chi$, as can be seen in Fig. 8a. No significant difference in $\chi$ was observed with and without $\text {USP}_{\text {EL}}$, near and in the full assembly for the three systems, for the frequency $f=3$ Hz studied in this work. To confirm this, the displacement of each (sphere) particle at the peak load at the $200{\text {th}}$ cycle is also plotted in Fig. 8 (middle and bottom row) for all cases. When looking at the particle displacement behavior, it is clear that the particle movement is more or less the same with and without $\text {USP}_{\text {EL}}$ for the three systems. Difference in the displacement field (and thus $\chi$) with and without $\text {USP}_{\text {EL}}$ is expected at higher loading frequencies, which is beyond the scope of the present study. When elastic layer at the bottom is used, high $\chi$ values for Kieselkalk in steel box with $\text {UBM}_{\text {EL}}$ was observed, as seen in Fig. 8b. High $\chi$ signifies high ballast movement, which can be seen the displacement field in Fig. 8 (second column), in agreement with the experimental observations of Raymond and Bathurst [22].

Regarding different ballast types, Calcite shows a slightly higher $\chi$ than Kieselkalk, plotted in Fig. 8a, c respectively. The difference is more pronounced when looking at the particle movement close to the sleeper. It is important to mention here that this difference in particle movement for the two types of ballast is also linked with the difference in the initial ballast packing after the preparation step. Higher movement for Kieselkalk compared to Calcite was also seen during repetition experiments (data not shown).

#### Macroscopic settlement behavior based on microscopic observations

Since, the contribution of edge breakage and particle movement during the loading cycles was clearly identified for all the systems, it is easy to spot the dominant mechanism for different sleeper settlement observed in Fig. 6 for:

(i) elastic layers at sleeper/ballast interface: the sleeper settlement with $\text {USP}_{\text {EL}}$ is smaller for the three systems as shown in Fig. 6. This is because all the sleeper/ballast contacts are yielding (edge breakage) with concrete sleeper, while no yielding can be seen at this interface when using $\text {USP}_{\text {EL}}$, as shown in Fig. 7a, b. Particle movement and yielding of the contacts in the full assembly have no major role on the observed lower settlement with $\text {USP}_{\text {EL}}$ for the three systems. Thus, the main source for smaller sleeper settlement with $\text {USP}_{\text {EL}}$ is the smaller edge breakage at the sleeper/ballast interface. Interestingly, the lower sleeper settlement with $\text {USP}_{\text {EL}}$ observed in the DEM simulations is in agreement with the experimental work by Berghold [10], Safari Baghsorkhi et al. [16], Gräbe et al. [18]. Though the number of loading cycles applied in this work is much smaller than the experimental references, the behavior of smaller settlement with $\text {USP}_{\text {EL}}$ is likely to follow the same trend for large loading cycles in DEM simulations.
(ii) elastic layers at ballast/bottom interface: Kieselkalk in steel box with $\text {UBM}_{\text {EL}}$ shows higher sleeper settlement compared to stiff steel bottom, as plotted in Figs. 6a, b. Edge breakage with $\text {UBM}_{\text {EL}}$ is approximately the same at sleeper/ballast interface, and higher by $2\%$ in the full assembly. Particle movement in the full system is substantially higher with $\text {UBM}_{\text {EL}}$ and is the main contributor for higher sleeper settlement observed with $\text {UBM}_{\text {EL}}$. Interestingly, the higher sleeper settlement observed with $\text {UBM}_{\text {EL}}$ is in agreement with the observation of Raymond and Bathurst [22], where high settlement was also observed for soft bottom made of rubber mats compared to stiff concrete bottom. Note that, though the use of $\text {UBM}_{\text {EL}}$ sounds counter-intuitive, they can be useful in reducing ballast fouling (by mitigating inflow of subgrade particles in the ballast bed), vibration isolation, stiffness adjustment in transition zones and in bridges/tunnels etc., which is not the objective of the present study.
(iii) different ballast types Calcite shows slightly higher sleeper settlement compared to Kieselkalk in steel box, as shown in Fig. 6a, c. Both types of ballast have shown similar level of edge breakage at the sleeper/ballast interface and in the full system, for both with and without $\text {USP}_{\text {EL}}$. Particle movement in the full system is slightly smaller for Kieselkalk than Calcite, as shown in Fig. 8a, c and is the primary reason for higher sleeper settlement observed for Calcite. The authors performed repeatability experiments with different random initial particle position followed by the same preparation procedure as described in Sect. 3. For some systems, Calcite showed slightly lower settlement compared to Kieselkalk. During the microscopic investigations, again a similar level of yielding was seen for the two types of ballast as presented here. However, opposite trend in the particle movements was observed in those experiments.

It is important to emphasize again the importance of correctly modelling the physical phenomena at the ballast scale in the DEM simulations – in the present work edge breakage through CDM law – can help to better understand and gain important insights into the microscopic mechanisms responsible for the macroscopic behavior of ballast. For completeness, cyclic loading behavior of Kieselkalk ballast with Hertz–Mindlin model (by setting $\sigma _{\max }$ to very high value) was also conducted. Since the simple Hertz–Mindlin contact model does not account for the edge breakage mechanism observed for railway ballast, ‘same’ (initial) settlement behavior was observed with and without $\text {USP}_{\text {EL}}$, as expected.

### Stiffness

**Fig. 9:**
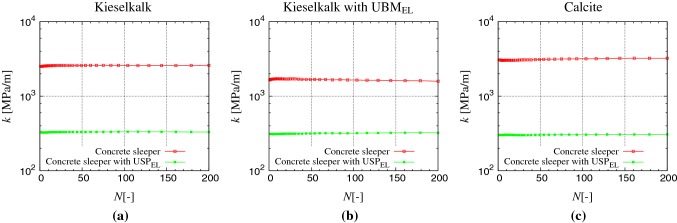
Stiffness $k_{{}}$ of the full system with cycles *N* for the three samples **a** Kieselkalk in steel box, **b** Kieselkalk in steel box with $\text {UBM}_{\text {EL}}$, and **c** Calcite in steel box. Red and green data sets represent results for the concrete sleeper and concrete sleeper with $\text {USP}_{\text {EL}}$ respectively (color figure online)

In this section, the influence of elastic layers and ballast types on trackbed stiffness is analyzed. The load–displacement curve of sleeper ballast assembly is typically non-linear and (hysteresis) dissipative in nature [13, 36]. Since the unloading branch is less sensitive to the deformation protocol and rate of deformation [34], the stiffness $k_{{}}$ here is calculated as the inverse ratio of the sleeper displacement between the two characteristic loads of 166 and 277 kPa during unloading. Note that, here the sleeper area is also accounted for calculating $k_{{}}$ to make an easy comparison and extension of the presented results to sleepers of different sizes.

Figure 9a shows the stiffness $k_{{}}$ with cycles *N* for Kieselkalk ballast in a steel box, with and without $\text {USP}_{\text {EL}}$. $k_{{}}$ is fairly constant during the 200 load cycles for both cases. Generally, with increasing number of cycles, ballast deterioration (widening of particle size distribution) and compaction of the bed [16] occurs, which in turn increases the system stiffness *k*, i.e., mechanical strength of the material [37]. However, performing DEM simulations to large cycles is beyond the scope of this work. Under the same loading conditions, large sleeper displacement were observed with $\text {USP}_{\text {EL}}$ compared to with concrete sleeper (see Fig. 5). Therefore, the trackbed stiffness $k_{{}}$ for Kieselkalk in a steel box with $\text {USP}_{\text {EL}}$ is smaller than without $\text {USP}_{\text {EL}}$, 330 MPa/m and 2580 MPa/m respectively. Smaller system stiffness with $\text {USP}_{\text {EL}}$ is in agreement with the experimental observation by [16].

Figure 9b shows the stiffness $k_{{}}$ of the Kieselkalk sample in a steel box with $\text {UBM}_{\text {EL}}$. The average $k_{{}}$ with and without $\text {USP}_{\text {EL}}$ are 320 and 1640 MPa/m respectively. Stiffness $k_{{}}$ for Calcite in a steel box is presented in Fig. 9c. Similar to Kieselkalk, $k_{{}}$ of Calcite assembly is seemingly constant during the 200 loading cycles. The average $k_{{}}$ for Calcite in the steel box, with and without $\text {USP}_{\text {EL}}$ are 305 MPa/m and 3240 MPa/m, respectively. The ratio of $k_{{}}$ for Calcite to Kieselkalk in a steel box without $\text {USP}_{\text {EL}}$ is 1.26. Interestingly, this value is quite close to the value 1.27, measured by Suhr et al. [1], where a steel plate fully covering the top of the box was used to apply compression. A summary of the stiffness $k_{{}}$ for the six different cases is presented in Table 3.

Next, the effects of elastic layers positioned at different locations in the ballast bed, on net $k_{{}}$ is examined for Kieselkalk ballast, by comparing Fig. 9a, b. When no elastic layers are used at the bottom, $k_{{}}$ reduces when an elastic layer $\text {USP}_{\text {EL}}$ is introduced at the sleeper/ballast interface. Similarly, for concrete sleeper without elastic layers at the sleeper/ballast interface, $k_{{}}$ reduces when $\text {UBM}_{\text {EL}}$ is introduced at the ballast-bottom interface. These observations have to be expected, since the smallest interaction stiffness between different track components, dominate the equivalent trackbed stiffness [38]. When elastic layer $\text {USP}_{\text {EL}}$ is used, the net $k_{{}}$ is approximately the same with and without $\text {UBM}_{\text {EL}}$ 320 MPa/m and 330 MPa/m respectively. This is to say that for the parameters of elastic layers used in this study, $k_{{}}$ is more or less independent of $\text {UBM}_{\text {EL}}$, when $\text {USP}_{\text {EL}}$ is used. The contact network of the ballast inside the bulk is approximately the same for all cases. Thus, the net $k_{{}}$ of the system is mainly governed by the number and stiffness of the contact that contribute to transfer the load at the sleeper/ballast and ballast/bottom interfaces. The contact springs are connected in parallel at these interfaces. The elastic layers, $\text {UBM}_{\text {EL}}$ and $\text {USP}_{\text {EL}}$, have approximately the same Young’s modulus, given in Table 1. On the other hand, the number of contacts at the sleeper/ballast interface is much smaller compared to at the ballast/bottom interface. As a result, the net $k_{{}}$ is governed by the (smallest) net stiffness of the sleeper/ballast interface with $\text {USP}_{\text {EL}}$. Thus, when $\text {USP}_{\text {EL}}$ are used, $k_{{}}$ for Kieselkalk in stiff bottom is more or less same (3% difference) as with elastic $\text {UBM}_{\text {EL}}$. This means that elastic layer under the sleeper – when chosen with the appropriate material property – may help in reducing the fluctuations in the stiffness along the track due to unavoidable variations in subgrade.

### Pressure distribution at the bottom

**Fig. 10:**
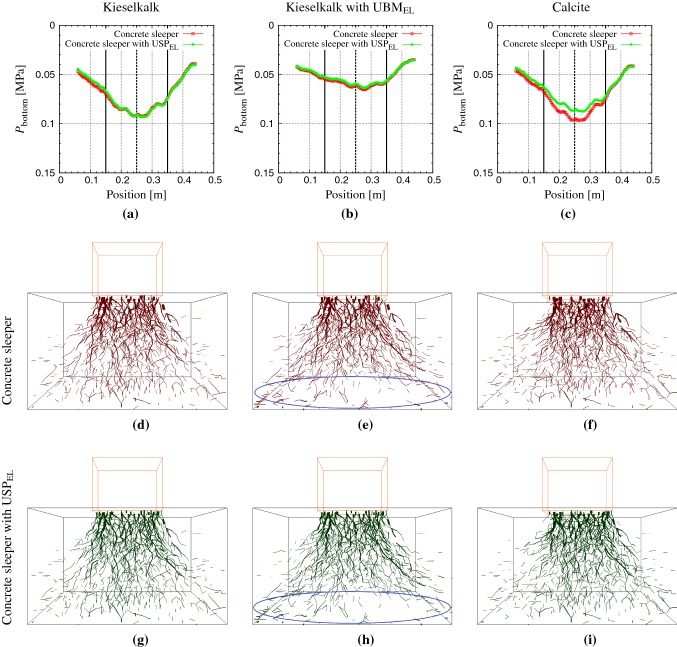
(Top row) Pressure distribution at the bottom of box at the peak load at the $200{\text {th}}$ cycle for the three samples: Kieselkalk in steel box (first column), Kieselkalk in steel box with $\text {UBM}_{\text {EL}}$ (second column), and Calcite in steel box (third column). Red and green data sets represent results for the concrete sleeper and concrete sleeper with $\text {USP}_{\text {EL}}$ respectively. For comparison purposes, same axes are used for all cases. Screenshots of the contact force chains in the normal direction at the peak load at the $200{\text {th}}$ cycle for systems with concrete sleeper (middle row) and concrete sleeper with $\text {USP}_{\text {EL}}$ (bottom row) for the three samples are shown in **d**–**i**. Line thickness is proportional to the contact force. Only contact forces $F_N>$ 70 N are plotted (color figure online)

In this section, the effects of elastic layers and ballast types on the pressure profile at the bottom of the box is investigated. Firstly, it is essential to describe the methodology used to measure the pressure. Though the pressure profile at the bottom is a 3D surface plot, only the pressure profile along the center lines of the box bottom is investigated. All the ballast/bottom contacts inside an imaginary squared bin of dimension 0.16 m $\times$ 0.16 m lying on the box bottom are recorded, and the pressure inside this bin is simply the sum of all the contact forces divided by the area of the bin. The imaginary squared bin is moved in small increments along the center line of the box bottom, parallel to *x* and *y*-axis, and pressure in each bin with position is calculated in both *x* and *y*-directions. There are around 40 ballast/bottom contacts in each bin. The average of the two directions is the bottom pressure $P_{\text {bottom}}$, plotted in Fig. 10 for all the cases described in Table 2, at the maximum load at the $200{\text {th}}$ cycle. The weight of the sleeper and ballast are excluded to make the figure easier to read; these are the additional forces required to maintain equilibrium.

The pressure for Kieselkalk ballast in a steel box with and without $\text {USP}_{\text {EL}}$ (cases I and II in Table 2) is shown in Fig. 10a. For both cases, as expected the pressure is maximum directly underneath the center of the sleeper and decreases away from the center. The pressure profile is symmetric around the center. This means that the force chains go downward homogeneously, and have no preferential direction, as shown in the corresponding force chain plot in Fig. 10d. Considering the effect of the elastic layer at the sleeper/ballast interface, only a marginal difference was observed in the pressure profile with and without $\text {USP}_{\text {EL}}$. This is confirmed when looking at the contact force chains in Fig. 10 (first column) for the two cases. In the deeper regions of the ballast bed, no significant influence of $\text {USP}_{\text {EL}}$ on the force chains was observed. Therefore, the pressure profile is almost the same, for both with and without $\text {USP}_{\text {EL}}$. This behavior is in agreement with the experimental work from [10, 20], where no significant difference was observed in contact pressure due to $\text {USP}_{\text {EL}}$. A summary of the average force in different sections of the ballast bed in given in Table 3 for the different cases considered in this study.

Regarding the effects of $\text {UBM}_{\text {EL}}$, Kieselkalk in a steel box with $\text {UBM}_{\text {EL}}$ shows a lower pressure $P_{\text {bottom}}$, when comparing Fig. 10a, b. Both the systems undergo the same loading condition, which means, if measured $P_{\text {bottom}}$ is small, forces should be transmitted laterally to the side walls. First and third column in Fig. 10 show the force chains for Kieselkalk in a steel box and Kieselkalk in a steel box with $\text {UBM}_{\text {EL}}$, respectively. When the two systems are compared in the center region of the box, the force chain look the same for the two cases. However, near to the bottom surface, compared to many force chains without $\text {UBM}_{\text {EL}}$, only few force chains with $\text {UBM}_{\text {EL}}$ can be seen (comparing Fig. 10d with Fig. 10e for concrete sleeper and Fig. 10g with Fig.10h for concrete sleeper with $\text {USP}_{\text {EL}}$). Forces in large numbers were observed on the side walls near to the bottom with $\text {UBM}_{\text {EL}}$. This is why the pressure measured at the bottom $P_{\text {bottom}}$ is smaller with $\text {UBM}_{\text {EL}}$ due to diffusion of the load laterally in the regions near to the bottom of the box. Further microscopic investigations must be carried out to understand better the lateral force transmission near to the bottom wall with $\text {UBM}_{\text {EL}}$.

Comparing the two types of ballast, Kieselkalk and Calcite ballast in a steel box show a similar bottom pressure profile plotted in Fig. 10a, c (cases I–IV in Table 2). This is confirmed when looking at the corresponding force chains plots, where no significant differences were seen the deeper regions in the first and second column in Fig. 10. This is to say that, the type of ballast and sleeper type (with and without $\text {USP}_{\text {EL}}$), do not have a big influence on the measured pressure at the bottom, in agreement with experimental work by  Abadi et al. [20].

## Conclusions and outlook

In this study, DEM simulations are conducted to investigate the effects of elastic layers on two different types of ballast: Kieselkalk and Calcite. Based on literature review, it was found that the initial settlement occurs at a fast rate, where ballast rearrangement and edge breakage are the dominant sources. The two physical mechanisms are simulated in the present work, and calibrated parameters of CDM model accounting for the edge breakage, are used from Suhr et al. [1] for the two types of ballast: Kieselkalk and Calcite. The goal of this work is to investigate the effects of elastic layers (under sleeper pad ($\text {USP}_{\text {EL}}$) at the sleeper/ballast interface and under ballast mat ($\text {UBM}_{\text {EL}}$) at the ballast/bottom interface) and ballast types on the micro-macro ballast behavior of ballast. All the different cases studied underwent the same loading path for 200 cycles. DEM simulations show that both, the contact area and the number of contacts at the sleeper/ballast interface increase with $\text {USP}_{\text {EL}}$. On the other hand, the number of contacts in the deeper regions of ballast bed were not affected by the $\text {USP}_{\text {EL}}$. DEM simulations confirm reduced edge breakage at the sleeper/ballast interface causing reduced settlement with $\text {USP}_{\text {EL}}$. During the cyclic loading over 200 cycles, different settlement was observed for the six different cases and following conclusions were drawn for:

(i) elastic layers at sleeper/ballast interface: $\text {USP}_{\text {EL}}$ reduced the settlement for all the cases considered – main mechanism is the significant reduction in edge breakage at the sleeper/ballast interface;
(ii) elastic layers at ballast/bottom interface: $\text {UBM}_{\text {EL}}$ increases the settlement for Kieselkalk ballast – main mechanism is high ballast movement throughout the system;
(iii) different ballast types: for the presented systems, Calcite showed slightly higher sleeper settlement compared to Kieselkalk – main mechanism is the higher movement for Calcite. In this context, initial packing of the ballast plays an important role and can assist in higher sleeper settlement for one type of ballast than the other.

Stiffness measured from the load–displacement curve is shown to be dependent on the properties of the individual track component. Elastic layers reduce the stiffness of the assembly, since they are the softest track components interacting with the ballast. The pressure measured at the bottom of the box was found to be independent of the ballast type and $\text {USP}_{\text {EL}}$, while the peak pressure reduced adequately with $\text {UBM}_{\text {EL}}$.

The application of the CDM model including particle edge breakage shows a very good agreement with the several experimental observations from the literature regarding the influence of different types of ballast and elastic layers. This is not possible when using the simple Hertz–Mindlin contact law where edge breakage is not considered. Thus, the application of the CDM model allow to gain a deep insight into microscopic phenomena occurring in the ballast which is the basis to improve the macroscopic system behavior by introducing new and adapting existing track components. In future, further microscopic investigations will be performed to understand better the effects of elastic layer on vibrations and oscillations of the ballast at high loading frequencies.

## Footnotes

1 Lateral diffusion of the load can also contribute to the different sleeper settlement, as shown for a single sleeper-ballast assembly in Li and McDowell [4]. In the present study, the force chains in the deeper regions of the ballast bed were only weakly affected by $\text {USP}_{\text {EL}}$, discussed later in Sect. 4.5. Therefore, the effect of lateral force distribution for assessing the dominant mechanisms responsible for the observed different sleeper settlement will not considered.
